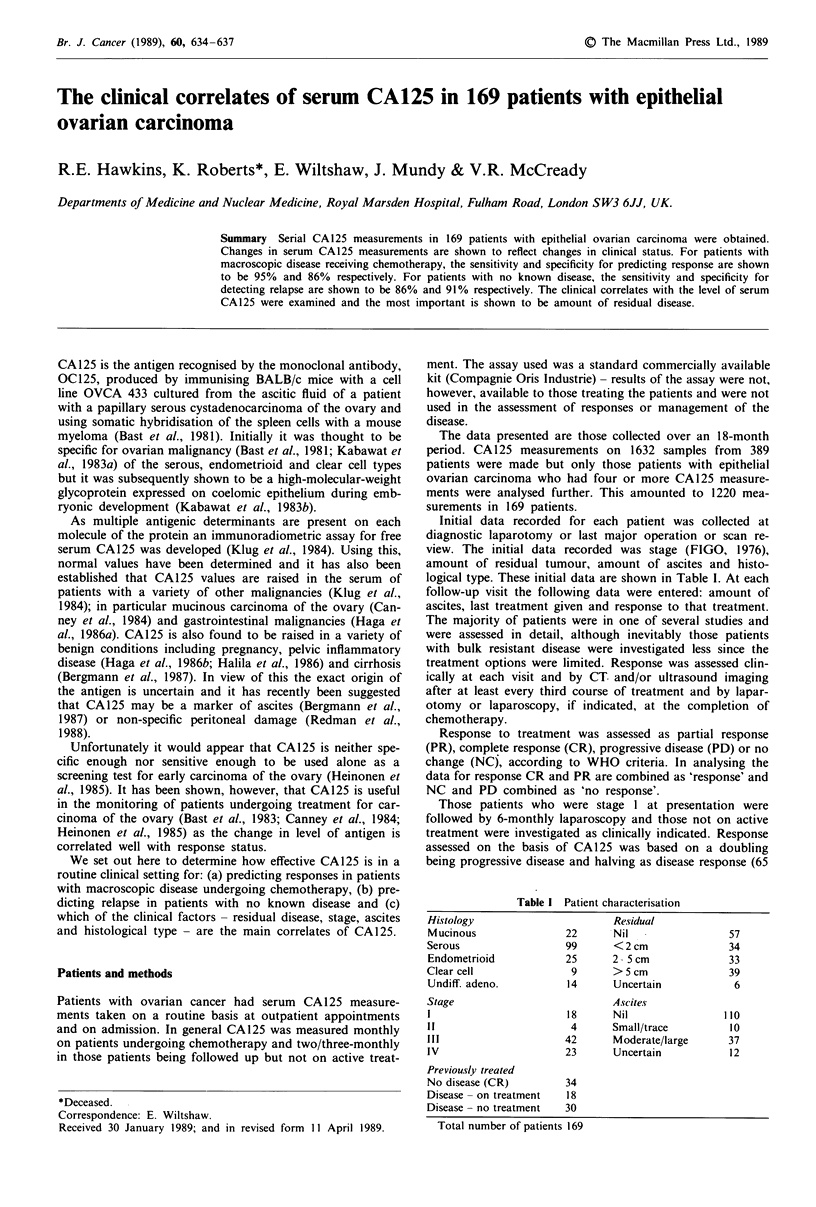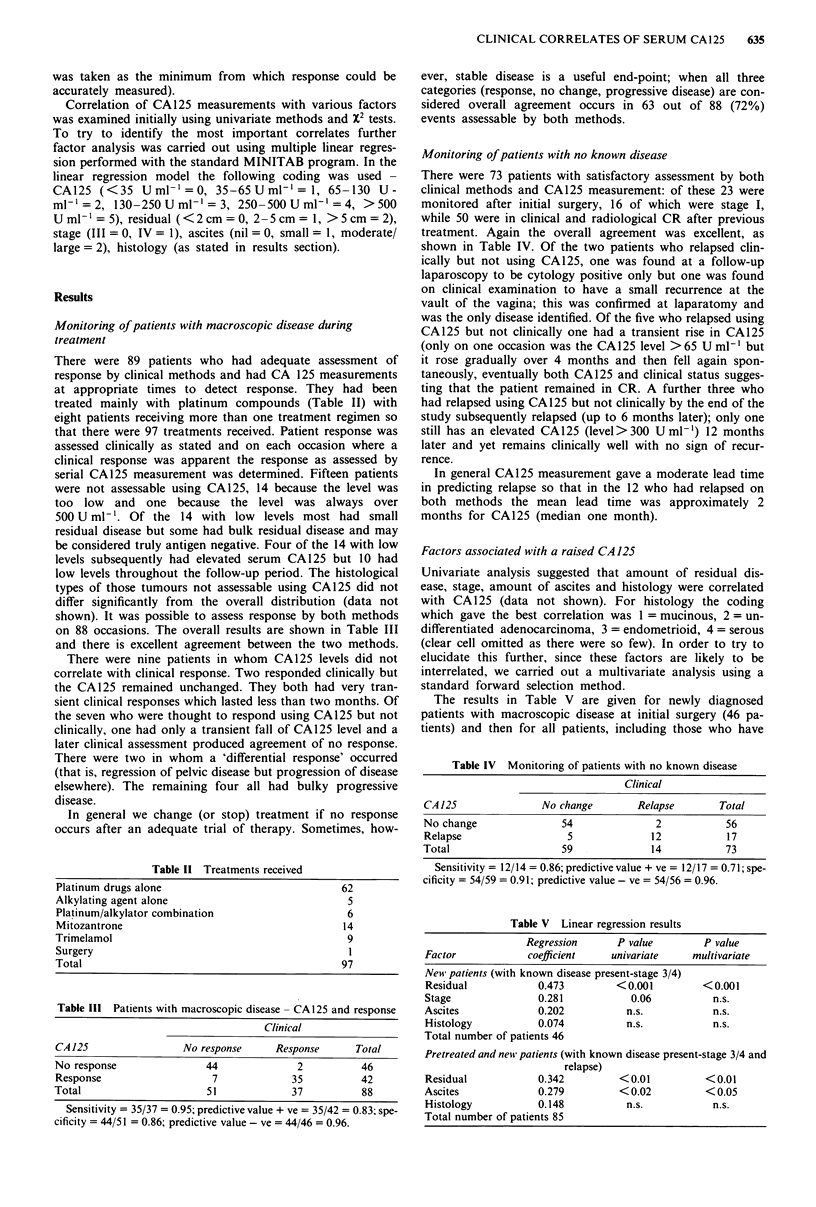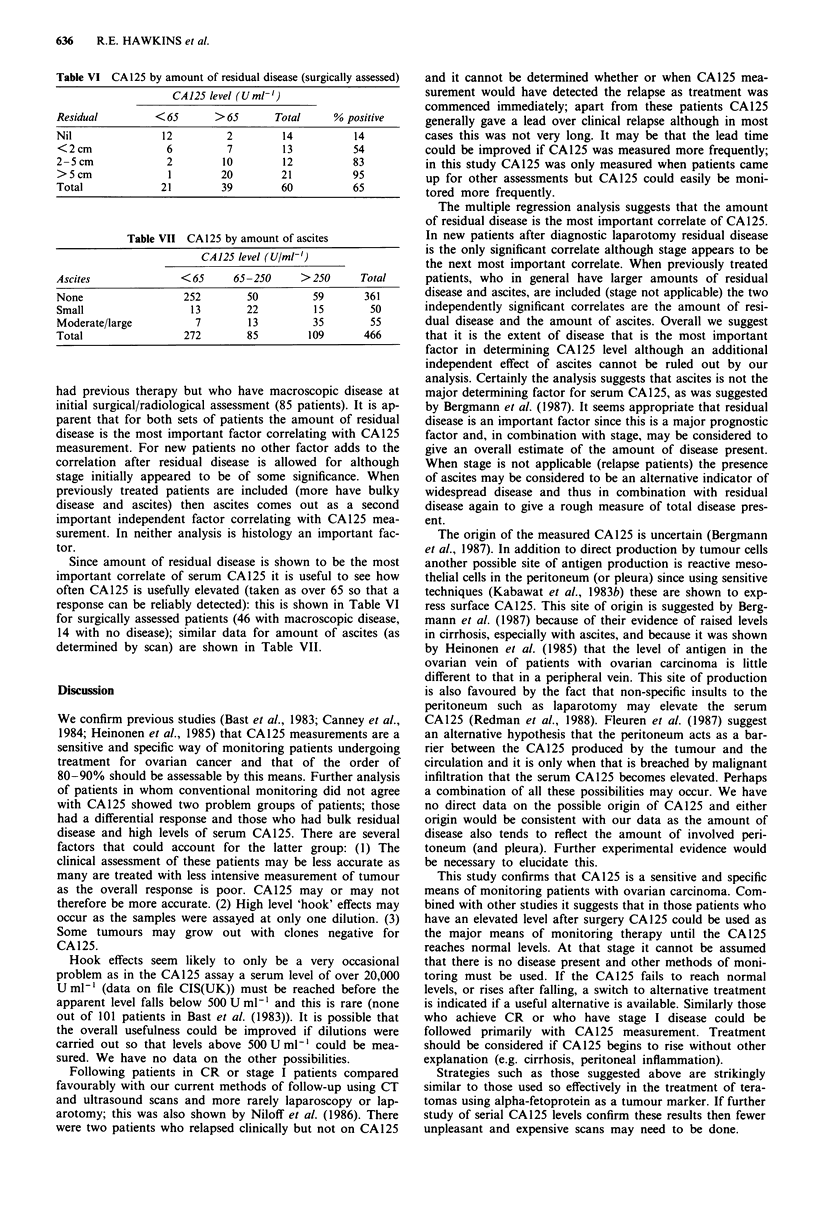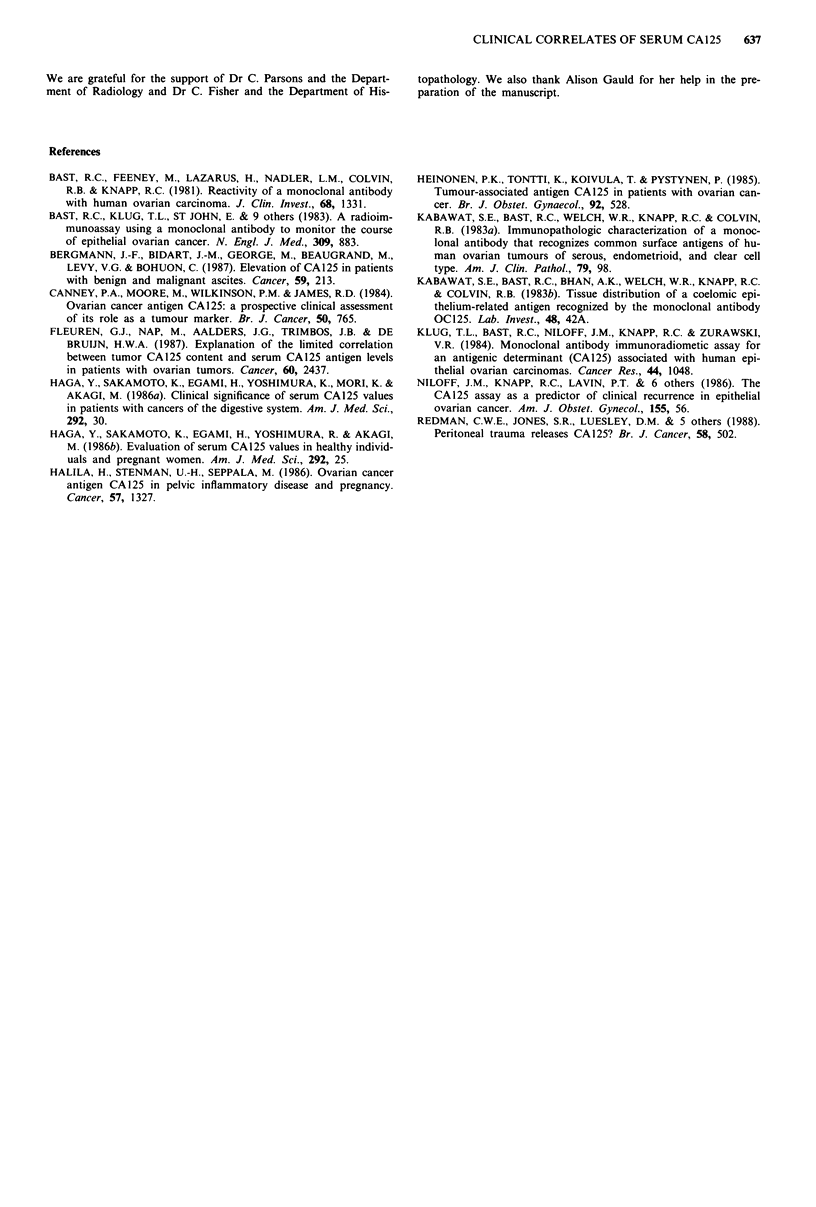# The clinical correlates of serum CA125 in 169 patients with epithelial ovarian carcinoma.

**DOI:** 10.1038/bjc.1989.329

**Published:** 1989-10

**Authors:** R. E. Hawkins, K. Roberts, E. Wiltshaw, J. Mundy, V. R. McCready

**Affiliations:** Department of Medicine, Royal Marsden Hospital, London, UK.

## Abstract

Serial CA125 measurements in 169 patients with epithelial ovarian carcinoma were obtained. Changes in serum CA125 measurements are shown to reflect changes in clinical status. For patients with macroscopic disease receiving chemotherapy, the sensitivity and specificity for predicting response are shown to be 95% and 86% respectively. For patients with no known disease, the sensitivity and specificity for detecting relapse are shown to be 86% and 91% respectively. The clinical correlates with the level of serum CA125 were examined and the most important is shown to be amount of residual disease.


Br. J. Cancer (1989), 60, 634-637 © The Macmillan Press Ltd., 1989

### The clinical correlates of serum CA125 in 169 patients with epithelial ovarian carcinoma

R.E. Hawkins, K. Roberts*, E. Wiltshaw, J. Mundy & V.R. McCready

*Departments of Medicine and Nuclear Medicine, Royal Marsden Hospital, Fulham Road, London SW3 6JJ, UK.*

**Summary** Serial CA125 measurements in 169 patients with epithelial ovarian carcinoma were obtained. Changes in serum CA125 measurements are shown to reflect changes in clinical status. For patients with macroscopic disease receiving chemotherapy, the sensitivity and specificity for predicting response are shown to be 95% and 86% respectively. For patients with no known disease, the sensitivity and specificity for detecting relapse are shown to be 86% and 91% respectively. The clinical correlates with the level of serum CA125 were examined and the most important is shown to be amount of residual disease.

CA125 is the antigen recognised by the monoclonal antibody, OC125, produced by immunising BALB/c mice with a cell line OVCA 433 cultured from the ascitic fluid of a patient with a papillary serous cystadenocarcinoma of the ovary and using somatic hybridisation of the spleen cells with a mouse myeloma (Bast *et al.*, 1981). Initially it was thought to be specific for ovarian malignancy (Bast *et al.*, 1981; Kabawat *et al.*, 1983*a*) of the serous, endometrioid and clear cell types but it was subsequently shown to be a high-molecular-weight glycoprotein expressed on coelomic epithelium during embryonic development (Kabawat *et al.*, 1983*b*).

As multiple antigenic determinants are present on each molecule of the protein an immunoradiometric assay for free serum CA125 was developed (Klug *et al.*, 1984). Using this, normal values have been determined and it has also been established that CA125 values are raised in the serum of patients with a variety of other malignancies (Klug *et al.*, 1984); in particular mucinous carcinoma of the ovary (Canney *et al.*, 1984) and gastrointestinal malignancies (Haga *et al.*, 1986*a*). CA125 is also found to be raised in a variety of benign conditions including pregnancy, pelvic inflammatory disease (Haga *et al.*, 1986*b*; Halila *et al.*, 1986) and cirrhosis (Bergmann *et al.*, 1987). In view of this the exact origin of the antigen is uncertain and it has recently been suggested that CA125 may be a marker of ascites (Bergmann *et al.*, 1987) or non-specific peritoneal damage (Redman *et al.*, 1988).

Unfortunately it would appear that CA125 is neither specific enough nor sensitive enough to be used alone as a screening test for early carcinoma of the ovary (Heinonen *et al.*, 1985). It has been shown, however, that CA125 is useful in the monitoring of patients undergoing treatment for carcinoma of the ovary (Bast *et al.*, 1983; Canney *et al.*, 1984; Heinonen *et al.*, 1985) as the change in level of antigen is correlated well with response status.

We set out here to determine how effective CA125 is in a routine clinical setting for: (a) predicting responses in patients with macroscopic disease undergoing chemotherapy, (b) predicting relapse in patients with no known disease and (c) which of the clinical factors – residual disease, stage, ascites and histological type – are the main correlates of CA125.

### Patients and methods

Patients with ovarian cancer had serum CA125 measurements taken on a routine basis at outpatient appointments and on admission. In general CA125 was measured monthly on patients undergoing chemotherapy and two/three-monthly in those patients being followed up but not on active treatment. The assay used was a standard commercially available kit (Compagnie Oris Industrie) – results of the assay were not, however, available to those treating the patients and were not used in the assessment of responses or management of the disease.

The data presented are those collected over an 18-month period. CA125 measurements on 1632 samples from 389 patients were made but only those patients with epithelial ovarian carcinoma who had four or more CA125 measurements were analysed further. This amounted to 1220 measurements in 169 patients.

Initial data recorded for each patient was collected at diagnostic laparotomy or last major operation or scan review. The initial data recorded was stage (FIGO, 1976), amount of residual tumour, amount of ascites and histological type. These initial data are shown in Table I. At each follow-up visit the following data were entered: amount of ascites, last treatment given and response to that treatment. The majority of patients were in one of several studies and were assessed in detail, although inevitably those patients with bulk resistant disease were investigated less since the treatment options were limited. Response was assessed clinically at each visit and by CT and/or ultrasound imaging after at least every third course of treatment and by laparotomy or laparoscopy, if indicated, at the completion of chemotherapy.

Response to treatment was assessed as partial response (PR), complete response (CR), progressive disease (PD) or no change (NC), according to WHO criteria. In analysing the data for response CR and PR are combined as 'response' and NC and PD combined as 'no response'.

Those patients who were stage 1 at presentation were followed by 6-monthly laparoscopy and those not on active treatment were investigated as clinically indicated. Response assessed on the basis of CA125 was based on a doubling being progressive disease and halving as disease response (65

**Table I** Patient characterisation

| | | | |
|---|---|---|---|
| *Histology* | | *Residual* | |
| Mucinous | 22 | Nil | 57 |
| Serous | 99 | <2 cm | 34 |
| Endometrioid | 25 | 2-5 cm | 33 |
| Clear cell | 9 | >5 cm | 39 |
| Undiff. adeno. | 14 | Uncertain | 6 |
| *Stage* | | *Ascites* | |
| I | 18 | Nil | 110 |
| II | 4 | Small/trace | 10 |
| III | 42 | Moderate/large | 37 |
| IV | 23 | Uncertain | 12 |
| *Previously treated* | | | |
| No disease (CR) | 34 | | |
| Disease – on treatment | 18 | | |
| Disease – no treatment | 30 | | |

Total number of patients 169

*Deceased.
Correspondence: E. Wiltshaw.
Received 30 January 1989; and in revised form 11 April 1989.

was taken as the minimum from which response could be accurately measured).

Correlation of CA125 measurements with various factors was examined initially using univariate methods and $\chi^2$ tests. To try to identify the most important correlates further factor analysis was carried out using multiple linear regression performed with the standard MINITAB program. In the linear regression model the following coding was used – CA125 ($<35$ U ml$^{-1}=0$, 35–65 U ml$^{-1}=1$, 65–130 U ml$^{-1}=2$, 130–250 U ml$^{-1}=3$, 250–500 U ml$^{-1}=4$, $>500$ U ml$^{-1}=5$), residual ($<2$ cm $=0$, 2–5 cm $=1$, $>5$ cm $=2$), stage (III = 0, IV = 1), ascites (nil = 0, small = 1, moderate/large = 2), histology (as stated in results section).

### Results

### *Monitoring of patients with macroscopic disease during treatment*

There were 89 patients who had adequate assessment of response by clinical methods and had CA 125 measurements at appropriate times to detect response. They had been treated mainly with platinum compounds (Table II) with eight patients receiving more than one treatment regimen so that there were 97 treatments received. Patient response was assessed clinically as stated and on each occasion where a clinical response was apparent the response as assessed by serial CA125 measurement was determined. Fifteen patients were not assessable using CA125, 14 because the level was too low and one because the level was always over 500 U ml$^{-1}$. Of the 14 with low levels most had small residual disease but some had bulk residual disease and may be considered truly antigen negative. Four of the 14 with low levels subsequently had elevated serum CA125 but 10 had low levels throughout the follow-up period. The histological types of those tumours not assessable using CA125 did not differ significantly from the overall distribution (data not shown). It was possible to assess response by both methods on 88 occasions. The overall results are shown in Table III and there is excellent agreement between the two methods.

There were nine patients in whom CA125 levels did not correlate with clinical response. Two responded clinically but the CA125 remained unchanged. They both had very transient clinical responses which lasted less than two months. Of the seven who were thought to respond using CA125 but not clinically, one had only a transient fall of CA125 level and a later clinical assessment produced agreement of no response. There were two in whom a 'differential response' occurred (that is, regression of pelvic disease but progression of disease elsewhere). The remaining four all had bulky progressive disease.

In general we change (or stop) treatment if no response occurs after an adequate trial of therapy. Sometimes, however, stable disease is a useful end-point; when all three categories (response, no change, progressive disease) are considered overall agreement occurs in 63 out of 88 (72%) events assessable by both methods.

**Table II** Treatments received

| | |
|---|---|
| Platinum drugs alone | 62 |
| Alkylating agent alone | 5 |
| Platinum/alkylator combination | 6 |
| Mitozantrone | 14 |
| Trimelamol | 9 |
| Surgery | 1 |
| Total | 97 |

**Table III** Patients with macroscopic disease – CA125 and response

| | *Clinical* | | |
|---|---|---|---|
| *CA125* | *No response* | *Response* | *Total* |
| No response | 44 | 2 | 46 |
| Response | 7 | 35 | 42 |
| Total | 51 | 37 | 88 |

Sensitivity = 35/37 = 0.95; predictive value + ve = 35/42 = 0.83; specificity = 44/51 = 0.86; predictive value – ve = 44/46 = 0.96.

### *Monitoring of patients with no known disease*

There were 73 patients with satisfactory assessment by both clinical methods and CA125 measurement: of these 23 were monitored after initial surgery, 16 of which were stage I, while 50 were in clinical and radiological CR after previous treatment. Again the overall agreement was excellent, as shown in Table IV. Of the two patients who relapsed clinically but not using CA125, one was found at a follow-up laparoscopy to be cytology positive only but one was found on clinical examination to have a small recurrence at the vault of the vagina; this was confirmed at laparatomy and was the only disease identified. Of the five who relapsed using CA125 but not clinically one had a transient rise in CA125 (only on one occasion was the CA125 level $>65$ U ml$^{-1}$ but it rose gradually over 4 months and then fell again spontaneously, eventually both CA125 and clinical status suggesting that the patient remained in CR. A further three who had relapsed using CA125 but not clinically by the end of the study subsequently relapsed (up to 6 months later); only one still has an elevated CA125 (level $>300$ U ml$^{-1}$) 12 months later and yet remains clinically well with no sign of recurrence.

In general CA125 measurement gave a moderate lead time in predicting relapse so that in the 12 who had relapsed on both methods the mean lead time was approximately 2 months for CA125 (median one month).

### *Factors associated with a raised CA125*

Univariate analysis suggested that amount of residual disease, stage, amount of ascites and histology were correlated with CA125 (data not shown). For histology the coding which gave the best correlation was 1 = mucinous, 2 = undifferentiated adenocarcinoma, 3 = endometrioid, 4 = serous (clear cell omitted as there were so few). In order to try to elucidate this further, since these factors are likely to be interrelated, we carried out a multivariate analysis using a standard forward selection method.

The results in Table V are given for newly diagnosed patients with macroscopic disease at initial surgery (46 patients) and then for all patients, including those who have

**Table IV** Monitoring of patients with no known disease

| | *Clinical* | | |
|---|---|---|---|
| *CA125* | *No change* | *Relapse* | *Total* |
| No change | 54 | 2 | 56 |
| Relapse | 5 | 12 | 17 |
| Total | 59 | 14 | 73 |

Sensitivity = 12/14 = 0.86; predictive value + ve = 12/17 = 0.71; specificity = 54/59 = 0.91; predictive value – ve = 54/56 = 0.96.

**Table V** Linear regression results

| *Factor* | *Regression coefficient* | *P value univariate* | *P value multivariate* |
|---|---|---|---|
| *New patients* (with known disease present-stage 3/4) | | | |
| Residual | 0.473 | <0.001 | <0.001 |
| Stage | 0.281 | 0.06 | n.s. |
| Ascites | 0.202 | n.s. | n.s. |
| Histology | 0.074 | n.s. | n.s. |
| Total number of patients 46 | | | |
| *Pretreated and new patients* (with known disease present-stage 3/4 and relapse) | | | |
| Residual | 0.342 | <0.01 | <0.01 |
| Ascites | 0.279 | <0.02 | <0.05 |
| Histology | 0.148 | n.s. | n.s. |
| Total number of patients 85 | | | |

**Table VI** CA125 by amount of residual disease (surgically assessed)

| | *CA125 level (U ml⁻¹)* | | | |
|---|---|---|---|---|
| *Residual* | *<65* | *>65* | *Total* | *% positive* |
| Nil | 12 | 2 | 14 | 14 |
| <2 cm | 6 | 7 | 13 | 54 |
| 2–5 cm | 2 | 10 | 12 | 83 |
| >5 cm | 1 | 20 | 21 | 95 |
| Total | 21 | 39 | 60 | 65 |

**Table VII** CA125 by amount of ascites

| | *CA125 level (U/ml⁻¹)* | | | |
|---|---|---|---|---|
| *Ascites* | *<65* | *65–250* | *>250* | *Total* |
| None | 252 | 50 | 59 | 361 |
| Small | 13 | 22 | 15 | 50 |
| Moderate/large | 7 | 13 | 35 | 55 |
| Total | 272 | 85 | 109 | 466 |

had previous therapy but who have macroscopic disease at initial surgical/radiological assessment (85 patients). It is apparent that for both sets of patients the amount of residual disease is the most important factor correlating with CA125 measurement. For new patients no other factor adds to the correlation after residual disease is allowed for although stage initially appeared to be of some significance. When previously treated patients are included (more have bulky disease and ascites) then ascites comes out as a second important independent factor correlating with CA125 measurement. In neither analysis is histology an important factor.

Since amount of residual disease is shown to be the most important correlate of serum CA125 it is useful to see how often CA125 is usefully elevated (taken as over 65 so that a response can be reliably detected): this is shown in Table VI for surgically assessed patients (46 with macroscopic disease, 14 with no disease); similar data for amount of ascites (as determined by scan) are shown in Table VII.

### Discussion

We confirm previous studies (Bast *et al.*, 1983; Canney *et al.*, 1984; Heinonen *et al.*, 1985) that CA125 measurements are a sensitive and specific way of monitoring patients undergoing treatment for ovarian cancer and that of the order of 80–90% should be assessable by this means. Further analysis of patients in whom conventional monitoring did not agree with CA125 showed two problem groups of patients; those had a differential response and those who had bulk residual disease and high levels of serum CA125. There are several factors that could account for the latter group: (1) The clinical assessment of these patients may be less accurate as many are treated with less intensive measurement of tumour as the overall response is poor. CA125 may or may not therefore be more accurate. (2) High level 'hook' effects may occur as the samples were assayed at only one dilution. (3) Some tumours may grow out with clones negative for CA125.

Hook effects seem likely to only be a very occasional problem as in the CA125 assay a serum level of over 20,000 U ml$^{-1}$ (data on file CIS(UK)) must be reached before the apparent level falls below 500 U ml$^{-1}$ and this is rare (none out of 101 patients in Bast *et al.* (1983)). It is possible that the overall usefulness could be improved if dilutions were carried out so that levels above 500 U ml$^{-1}$ could be measured. We have no data on the other possibilities.

Following patients in CR or stage I patients compared favourably with our current methods of follow-up using CT and ultrasound scans and more rarely laparoscopy or laparotomy; this was also shown by Niloff *et al.* (1986). There were two patients who relapsed clinically but not on CA125 and it cannot be determined whether or when CA125 measurement would have detected the relapse as treatment was commenced immediately; apart from these patients CA125 generally gave a lead over clinical relapse although in most cases this was not very long. It may be that the lead time could be improved if CA125 was measured more frequently; in this study CA125 was only measured when patients came up for other assessments but CA125 could easily be monitored more frequently.

The multiple regression analysis suggests that the amount of residual disease is the most important correlate of CA125. In new patients after diagnostic laparotomy residual disease is the only significant correlate although stage appears to be the next most important correlate. When previously treated patients, who in general have larger amounts of residual disease and ascites, are included (stage not applicable) the two independently significant correlates are the amount of residual disease and the amount of ascites. Overall we suggest that it is the extent of disease that is the most important factor in determining CA125 level although an additional independent effect of ascites cannot be ruled out by our analysis. Certainly the analysis suggests that ascites is not the major determining factor for serum CA125, as was suggested by Bergmann *et al.* (1987). It seems appropriate that residual disease is an important factor since this is a major prognostic factor and, in combination with stage, may be considered to give an overall estimate of the amount of disease present. When stage is not applicable (relapse patients) the presence of ascites may be considered to be an alternative indicator of widespread disease and thus in combination with residual disease again to give a rough measure of total disease present.

The origin of the measured CA125 is uncertain (Bergmann *et al.*, 1987). In addition to direct production by tumour cells another possible site of antigen production is reactive mesothelial cells in the peritoneum (or pleura) since using sensitive techniques (Kabawat *et al.*, 1983*b*) these are shown to express surface CA125. This site of origin is suggested by Bergmann *et al.* (1987) because of their evidence of raised levels in cirrhosis, especially with ascites, and because it was shown by Heinonen *et al.* (1985) that the level of antigen in the ovarian vein of patients with ovarian carcinoma is little different to that in a peripheral vein. This site of production is also favoured by the fact that non-specific insults to the peritoneum such as laparotomy may elevate the serum CA125 (Redman *et al.*, 1988). Fleuren *et al.* (1987) suggest an alternative hypothesis that the peritoneum acts as a barrier between the CA125 produced by the tumour and the circulation and it is only when that is breached by malignant infiltration that the serum CA125 becomes elevated. Perhaps a combination of all these possibilities may occur. We have no direct data on the possible origin of CA125 and either origin would be consistent with our data as the amount of disease also tends to reflect the amount of involved peritoneum (and pleura). Further experimental evidence would be necessary to elucidate this.

This study confirms that CA125 is a sensitive and specific means of monitoring patients with ovarian carcinoma. Combined with other studies it suggests that in those patients who have an elevated level after surgery CA125 could be used as the major means of monitoring therapy until the CA125 reaches normal levels. At that stage it cannot be assumed that there is no disease present and other methods of monitoring must be used. If the CA125 fails to reach normal levels, or rises after falling, a switch to alternative treatment is indicated if a useful alternative is available. Similarly those who achieve CR or who have stage I disease could be followed primarily with CA125 measurement. Treatment should be considered if CA125 begins to rise without other explanation (e.g. cirrhosis, peritoneal inflammation).

Strategies such as those suggested above are strikingly similar to those used so effectively in the treatment of teratomas using alpha-fetoprotein as a tumour marker. If further study of serial CA125 levels confirm these results then fewer unpleasant and expensive scans may need to be done.

We are grateful for the support of Dr C. Parsons and the Department of Radiology and Dr C. Fisher and the Department of Histopathology. We also thank Alison Gauld for her help in the preparation of the manuscript.

### References

BAST, R.C., FEENEY, M., LAZARUS, H., NADLER, L.M., COLVIN, R.B. & KNAPP, R.C. (1981). Reactivity of a monoclonal antibody with human ovarian carcinoma. *J. Clin. Invest.*, **68**, 1331.

BAST, R.C., KLUG, T.L., ST JOHN, E. & 9 others (1983). A radioimmunoassay using a monoclonal antibody to monitor the course of epithelial ovarian cancer. *N. Engl. J. Med.*, **309**, 883.

BERGMANN, J.-F., BIDART, J.-M., GEORGE, M., BEAUGRAND, M., LEVY, V.G. & BOHUON, C. (1987). Elevation of CA125 in patients with benign and malignant ascites. *Cancer*, **59**, 213.

CANNEY, P.A., MOORE, M., WILKINSON, P.M. & JAMES, R.D. (1984). Ovarian cancer antigen CA125: a prospective clinical assessment of its role as a tumour marker. *Br. J. Cancer*, **50**, 765.

FLEUREN, G.J., NAP, M., AALDERS, J.G., TRIMBOS, J.B. & DE BRUIJN, H.W.A. (1987). Explanation of the limited correlation between tumor CA125 content and serum CA125 antigen levels in patients with ovarian tumors. *Cancer*, **60**, 2437.

HAGA, Y., SAKAMOTO, K., EGAMI, H., YOSHIMURA, K., MORI, K. & AKAGI, M. (1986*a*). Clinical significance of serum CA125 values in patients with cancers of the digestive system. *Am. J. Med. Sci.*, **292**, 30.

HAGA, Y., SAKAMOTO, K., EGAMI, H., YOSHIMURA, R. & AKAGI, M. (1986*b*). Evaluation of serum CA125 values in healthy individuals and pregnant women. *Am. J. Med. Sci.*, **292**, 25.

HALILA, H., STENMAN, U.-H., SEPPALA, M. (1986). Ovarian cancer antigen CA125 in pelvic inflammatory disease and pregnancy. *Cancer*, **57**, 1327.

HEINONEN, P.K., TONTTI, K., KOIVULA, T. & PYSTYNEN, P. (1985). Tumour-associated antigen CA125 in patients with ovarian cancer. *Br. J. Obstet. Gynaecol.*, **92**, 528.

KABAWAT, S.E., BAST, R.C., WELCH, W.R., KNAPP, R.C. & COLVIN, R.B. (1983*a*). Immunopathologic characterization of a monoclonal antibody that recognizes common surface antigens of human ovarian tumours of serous, endometrioid, and clear cell type. *Am. J. Clin. Pathol.*, **79**, 98.

KABAWAT, S.E., BAST, R.C., BHAN, A.K., WELCH, W.R., KNAPP, R.C. & COLVIN, R.B. (1983*b*). Tissue distribution of a coelomic epithelium-related antigen recognized by the monoclonal antibody OC125. *Lab. Invest.*, **48**, 42A.

KLUG, T.L., BAST, R.C., NILOFF, J.M., KNAPP, R.C. & ZURAWSKI, V.R. (1984). Monoclonal antibody immunoradiometic assay for an antigenic determinant (CA125) associated with human epithelial ovarian carcinomas. *Cancer Res.*, **44**, 1048.

NILOFF, J.M., KNAPP, R.C., LAVIN, P.T. & 6 others (1986). The CA125 assay as a predictor of clinical recurrence in epithelial ovarian cancer. *Am. J. Obstet. Gynecol.*, **155**, 56.

REDMAN, C.W.E., JONES, S.R., LUESLEY, D.M. & 5 others (1988). Peritoneal trauma releases CA125? *Br. J. Cancer*, **58**, 502.